# Determinants of clinical improvement after surgical replacement or transcatheter aortic valve implantation for isolated aortic stenosis

**DOI:** 10.1186/1476-7120-12-41

**Published:** 2014-10-06

**Authors:** Cristina Gavina, Alexandra Gonçalves, Carlos Almeria, Rosana Hernandez, Adelino Leite-Moreira, Francisco Rocha-Gonçalves, José Zamorano

**Affiliations:** Department of Medicine, Faculty of Medicine, University of Porto, Rua Dr. Plácido da Costa 4200-450, Porto, Portugal; Department of Cardiology, Hospital Clínico San Carlos, Madrid, Spain; Department of Physiology and Cardiothoracic Surgery, Faculty of Medicine, University of Porto, Kragujevac, Portugal; Department of Cardiothoracic Surgery, Centro Hospitalar São João, Porto, Portugal; Department of Cardiology, University Hospital Ramón y Cajal, Madrid, Spain

**Keywords:** Aortic stenosis, Transcatheter aortic valve replacement, Surgical aortic valve replacement, Left ventricular mass, Reverse remodeling, Clinical improvement

## Abstract

**Background:**

Transcatheter aortic valve implantation (TAVI) is an alternative to surgical aortic valve replacement (SAVR) in patients with aortic stenosis (AS) and high surgical risk. Hemodynamic performance after TAVI is superior, but the impact of reverse remodeling on clinical improvement is controversial. We aim to address the differences in hemodynamic changes between SAVR and TAVI, and its correlation with LV remodeling and clinical improvement at 6 months follow-up.

**Methods:**

Forty-two patients treated by TAVI were compared with 45 SAVR patients with a stented bioprosthesis. Clinical, 2D and 3D echocardiographic data were prospectively obtained before and six months after intervention.

**Results:**

Patients had similar distribution for sex, body surface area and AS severity. TAVI patients were older, more symptomatic and had more comorbidities. They also had higher LV filling pressures, larger 3D indexed left atrium volume, but similar 3D indexed LV mass. At 6 months, TAVI patients had greater clinical improvement and higher effective orifice area index (EAOI), but only SAVR patients already had a significant decrease in 3D indexed LV mass and diastolic volume. In univariate analysis older age, NYHA class ≥ III, increase in EAOI and TAVI were related with functional class improvement. After multivariate analysis only NYHA class ≥ III (OR 8.81, CI:2.13-36.52; p = 0.003) and an increase in EAOI ≥ 105% (OR 3.87, CI:1.02-14.70; p = 0.04) were predictors of clinical improvement.

**Conclusions:**

At 6 months, functional class improvement was greater after TAVI. Higher initial NYHA class and an increase in EAOI ≥ 105% were independently associated with functional enhancement. It is debatable if left ventricular remodeling is determinant for functional class improvement.

**Electronic supplementary material:**

The online version of this article (doi:10.1186/1476-7120-12-41) contains supplementary material, which is available to authorized users.

## Background

Aortic stenosis (AS) is the most prevalent of all valvular diseases in developed countries and its increase has a direct relation with population aging [1]. In elderly patients cardiac surgery can be challenging by the increased number of comorbidities, making transcatheter aortic valve implantation (TAVI) an atractive alternative treatment modality [2].

Surgical aortic valve replacement (SAVR) nearly normalizes long-term survival and improves quality of life in AS patients [3, 4] but late outcomes depend mainly on the stage of heart disease before surgery, prosthetic related complications, and comorbidities. Although there is a significant reduction of wall stress and left ventricular (LV) pressure after SAVR, nearly half of patients have residual LV hypertrophy (LVH) late after surgery [5, 6]. This persistent increase in LV mass is an independent predictor of cardiac-related morbidity [6] and mortality [7] making reverse remodeling an important outcome after surgery.

Similarly, TAVI has shown good mid-term results, not inferior to SAVR in high-risk patients [2] and superior to medical therapy [8]. TAVI patients have higher effective orifice areas (EOA) and lower transprosthetic gradients but, in spite of remarkable clinical improvement, LV mass regression and reverse remodeling are less consistent in comparison with SAVR [9–11].

In this study we aim to address the differences in hemodynamic changes between SAVR and TAVI patients, and its correlation with LV remodeling and clinical improvement at 6 months follow-up.

## Methods

### Patient selection and follow-up

The present study is a comparison of two prospective cohorts of patients with symptomatic degenerative severe AS (defined as aortic valve area ≤1 cm^2^) and LV ejection fraction (EF) ≥ 40%, who underwent SAVR with a stented bioprosthesis or TAVI. This was a collaborative work from two distinct institutions since one of them didn’t have a TAVI program at the time of patient inclusion.

Patients with aortic regurgitation > II/IV, moderate to severe mitral or tricuspid regurgitation, significant coronary artery disease (lesions >50% on coronary angiography) or previous cardiac surgery were excluded. All patients had a clinical and echocardiographic evaluation before and at 6 months after the procedure, if alive.

This investigation conforms to the Declaration of Helsinki, had institutional ethical review board approval and each study participant signed an informed consent before enrolment.

### TAVI group

Forty-two consecutive patients with severe aortic stenosis and preserved LV systolic function, submitted to successful TAVI in one tertiary center, Hospital Clinico San Carlos, from April 2009 to April 2010, were included. These patients were obtained from a series of 97 consecutive patients who underwent TAVI, after excluding those with significant LV systolic dysfunction, concomitant moderate to severe mitral valve disease or aortic regurgitation, and those with significant coronary artery disease.

Patients were referred for TAVI due to an excessive risk for SAVR, which was estimated using the logistic EuroSCORE and/or clinical judgment.

Vascular access was obtained either by percutaneous approach through the common femoral artery (30 patients) or transapical approach (12 patients).

The procedure was performed under fluoroscopy and transesophageal echocardiography (TEE) guidance using the techniques described in detail in previous reports [12]. Among all, 31 (73.8%) patients were implanted with an Edwards SAPIEN (Edwards Lifesciences, Irvine, CA, USA). The CoreValve (Medtronic CoreValve Percutaneous System, Medtronic CV) was implanted in 11 (26.2%) patients exclusively by retrograde transfemoral approach. Two valve sizes were available, 23- and 26-mm expanded diameter for Edwards SAPIEN valve and 26- and 29-mm for CoreValve. The prosthesis size was decided according to annulus diameter, measured by TEE. The deployment was performed under the agreement of the interventionist and the echocardiographer. Device success was defined as stable device placement and function as assessed by angiography and echocardiography. All patients with developing new grade III atrioventricular block were implanted with a permanent pacemaker within 3 days after valve implantation.

### SAVR group

Between January 2009 and December 2009, among 141 consecutive patients with isolated symptomatic AS referred for SAVR at the Cardiothoracic Surgery Department of Centro Hospitalar São João, Porto, Portugal we included 45 patients with 3D echocardiographic evaluation. All surgeries were performed using standard procedure for aortic valve replacement. Patients were placed on cardiopulmonary bypass and cardiac arrest was induced and maintained with cold blood cardioplegia. The prosthetic substitutes used in this study were Carpentier-Edwards Perimount pericardial valve (Edwards Lifesciences, Irvine, CA) and the St Jude Medical Epic porcine valve (St Jude Medical, Inc, St Paul, Minn). Valve sizes were 19 mm (n = 4;8.9%), 21 mm (n = 25;55.6%), 23 mm (n = 11;24.4%) and 25 mm (n = 5;11.1%).

### Echocardiographic studies

Echocardiographic examination was performed by a trained cardiologist and recorded on digital support. All recordings were examined by an experienced echocardiographer in an accredited independent echocardiography laboratory (Hospital Clínico San Carlos in Madrid, Spain), blinded to patient details. Studies were performed using Phillips iE-33 equipment with a S5-1 transducer with M-mode, two dimensional, pulsed, continuous, color-flow and tissue Doppler capabilities, and an X3-1 transducer for 3D imaging. Imaging analysis was performed with Xcelera and QLab software. All measurements were performed in accordance with the recommendations of the American Society of Echocardiography [13]. Peak transvalvular gradient was estimated using the simplified Bernoulli equation. Aortic valve area (or effective orifice area, EOA) was estimated using quantitative Doppler by the continuity equation. The EOA values were then indexed to body surface area (EAOI). Patient prosthesis mismatch was considered present if EAOI ≤0.85 cm2/m2 and severe when EAOI ≤ 0.65 cm2/m2.

The presence, degree, and type (paravalvular vs transvalvular) of aortic regurgitation (AR) were classified as trivial, mild, moderate, or severe according to The Valve Academic Research Consortium (VARC) II [14].

Mitral inflow was assessed in the apical 4-chamber view using pulsed wave Doppler with the sample volume placed at the tips of mitral leaflets during diastole. From the mitral inflow profile, the peak flow velocity of early filling (E wave), peak flow velocity of atrial contraction (A wave), the E/A ratio, and early filling deceleration time (DT) were measured. Doppler tissue imaging of the mitral annulus was obtained from the apical 4-chamber using a sample volume placed in the septal mitral valve annulus. The septal e’ velocity value was determined, and the E/e’ ratio was derived.

Left atrium (LA) volume, LV systolic and diastolic volumes, and resulting ejection fraction were calculated with direct 3D volumetric analysis.

All indexed values were obtained by dividing by body surface area according to the formula of Mosteler.

### Statistical analysis

Categorical variables were expressed as percentages and continuous variables as mean ± standard deviation or median and interquartile range, according to their distribution. Continuous variables were compared between groups using an unpaired *t*-test (for normally distributed variables) or the Mann–Whitney *U*-test (for non-normally distributed variables). For comparison between baseline and follow-up a paired Student's *t*-test was applied (normally distributed variables) and a Wilcoxon test (for non-normal distributed variables). Chi-square test was used to compare proportions. Following univariate analysis, a stepwise binary logistic multivariate regression model (Wald backward stepwise method, p = 0.05 for covariate inclusion and 0.1 for exclusion) was performed, including potential confounders for NYHA improvement regression analysis 6 months after AVR. NYHA improvement was analyzed as worse or equal vs better. Of note, patients in baseline NYHA class I but symptomatic (angina or syncope) were considered improved if these additional symptoms disappeared.

All reported probability values are two-tailed, and p < 0.05 was considered statistically significant. Analyses were performed with the IBM®SPSS® Statistics software package (version 21.0) (SPSS Inc, Chicago, IL, USA).

## Results

Patient’s baseline clinical characteristics are described in Table 1. TAVI patients were older, had more comorbidities and higher logistic Euroscore. Patients who underwent TAVI had worse functional class at baseline, although they had similar baseline severity of aortic stenosis (Table 2). Before intervention, when comparing to SAVR patients, TAVI patients had similar 3D LV mass index but, after normalizing LV mass to LV end-diastolic volume (LVMI/LVDVI), they had more concentric geometry (3.0 (P25-75:2.1-4.4) vs 2.4 (P25-75:1.8-3.0) g/ml; p = 0.044) due to smaller LV end-diastolic index volumes. TAVI patients also had worse diastolic dysfunction, with higher LV filling pressures and larger indexed LA volume (Table 3).

**Table 1:** **Baseline clinical characteristics of SAVR vs TAVI patients**

	SAVR (n = 45)	TAVI (n = 42)	p-value
**Age** **(years)** [Me (P25-P75)]	73 (68 - 78)	83.5 (79 - 87)	<0.001
**Female gender** [n(%)]	28 (62.2)	26 (61.9)	0.976
**BSA (** **m** ^**2**^ **)** [Me (P25-P75)]	1.76 (1.57 - 1.86)	1.75 (1.6 - 1.8)	0.639
**Hypertension** [n(%)]	28 (62.2)	35 (83.3)	0.028
**Diabetes mellitus** [n(%)]	13 (28.9)	11 (26.2)	0.778
**Dislipidemia** [n(%)]	28 (62.2)	26 (61.9)	0.976
**COPD** [n(%)]	12 (26.7)	19 (45.2)	0.071
**PAD** [n(%)]	3 (6.7)	12 (28.6)	0.007
**Atrial Fibrilation** [n(%)]	0 (0)	7 (16.7)	0.004
**Logistic Euroscore**	6.18 ± 2.71	17.86 ± 9.55	<0.001
**NYHA class III** **-** **IV** [n(%)]	12 (26.7)	37 (88.1)	<0.001

BSA = body surface area; COPD = chronic obstructive pulmonary disease; PAD = peripheral artery disease; NYHA = New York Heart Association; values are mean ± SD or median (P25-P75) according to distribution, or n (%).

**Table 2:** **Baseline and 6 months 2D echocardiographic data on SAVR vs TAVI patients**

	SAVR (n = 45)		TAVI (n = 42)		SAVR vs TAVIp-value
	mean ± SD	Me (P25-P75)	mean ± SD	Me (P25-P75)	
**AV annulus** (cm)					
Baseline	21.34 ± 2.18	21 (20 - 22.75)	20.73 ± 2.39	21 (19 - 22)	0.337^b^
**AV peak velocity** (cm/sec)					
Baseline	4.81 ± 0.60	4.76 (4.36 - 5.13)	4.76 ± 0.61	4.81 (4.36 - 5.16)	0.909^b^
6 months	2.69 ± 0.74	2.51 (2.21 - 3.00)	2.07 ± 0.51	2.11 (1.72 - 2.31)	<0.001^a^
p-value*	<0.001		<0.001		
**AV mean gradient** (mmHg)					
Baseline	57.89 ± 13.91	54.9 (47.13 - 66.45)	54.67 ± 15.77	52.5 (44.0 - 60.3)	0.317^a^
6 months	17.21 ± 12.05	13.9 (11 - 21)	8.86 ± 4.72	8.1 (5.7 - 11.4)	<0.001^a^
p-value**	<0.001		<0.001		
**AVA** (EOA, cm^2^)					
Baseline	0.69 ± 0.2	0.7 (0.52 - 0.83)	0.62 ± 0.15	0.6 (0.51 - 0.7)	0.070^a^
6 months	1.5 ± 0.42	1.4 (1.2 - 1.75)	1.95 ± 0.54	1.8 (1.5 - 2.2)	<0.001^a^
p-value**	<0.001		<0.001		
**AVA index** (EAOI cm^2^/m^2^)					
Baseline	0.40 ± 0.11	0.38 (0.32 - 0.47)	0.37 ± 0.1	0.37 (0.31 - 0.42)	0.229^b^
6 months	0.87 ± 0.24	0.82 (0.68 - 1.00)	1.16 ± 0.39	1.05 (0.88 - 1.36)	<0.001^a^
p-value*	<0.001		<0.001		
**Δ EAOI** (cm^2^/m^2^)	0.47 ± 0.28	0.46 (0.3 - 0.59)	0.79 ± 0.37	0.65 (0.55 - 1.03)	<0.001^a^
**PPM** [n (%)]	24 (58.6)		9 (23.1)		<0.001
**Severe PPM** [n (%)]	7 (17.1)		0		0.012
**Paravalvular AR** 6m [n (%)]	3 (6.7)		23 (59)		<0.001
**E/** **A ratio**					
Baseline	0.79 ± 0.34	0.72 (0.61 - 0.89)	1.38 ± 0.87	1.07 (0.74 - 1.74)	<0.001^b^
6 months	0.85 ± 0.25	0.81 (0.69 - 0.93)	1.34 ± 1.22	0.73 (0.57 - 1.74)	0.703^b^
p-value*	0.044		0.212		
**E** **-** **wave deceleration time** (ms)					
Baseline	235.11 ± 73.97	240 (180 - 280)	207.69 ± 79.97	198 (148 - 238.5)	0.039^b^
6 months	264.51 ± 72.84	250 (218.5 - 300)	251.82 ± 66.02	260 (190 - 295)	0.755^b^
p-value*	<0.001		<0.001		
**IVRT** (ms)					
Baseline	99.09 ± 26.85	100 (80 - 120)	73.4 ± 33.91	70 (50 - 100)	0.001^a^
6 months	116.43 ± 20.41	110 (100 - 130)	102.19 ± 28.93	100 (82.5 - 127.5)	0.034^a^
p-value**	0.049		<0.001		
**e’** (cm/s)					
Baseline	4.77 ± 1.8	4.4 (3.6 - 5.8)	5.56 ± 2.55	5 (4.1 - 6.1)	0.157^b^
6 months	5.59 ± 1.52	5.4 (4.6 - 6.73)	7 ± 3.07	6.1 (4.78 - 8.7)	0.033^a^
p-value*	0.005		0.495		
**E/** **e’**					
Baseline	18.62 ± 7.11	16.9 (13.27 - 23.68)	23.55 ± 10.88	25.36 (15.99 - 30.78)	0.024^b^
6 months	16.73 ± 5.96	15.75 (13.02 - 19.86)	16.36 ± 10.77	11.72 (8.33 - 23.06)	0.153^b^
p-value*	0.096		0.177		

AV = aortic valve; AVA = aortic valve area; EAOI = effective orifice area index; Δ EAOI = absolute increase in EAOI; PPM = patient-prosthesis mismatch; IVRT = Isovolumetric relaxation time; 6m = six months; values are mean ± SD or median (P25-P75) or n (%). ^a,b^ – different letters stand for significant differences in mean or median values according to *t*-test (a) or the Mann–Whitney *U* test (b).*Wilcoxon test; **Paired-sample *t*-test.

**Table 3:** **Baseline and 6 months 3D echocardiographic data on SAVR vs TAVI patients**

	SAVR (n = 45)		TAVI (n = 42)		SAVR vs TAVIp-value*
	mean ± SD	Me (P25-P75)	mean ± SD	Me (P25-P75)	
**LVEDVi** **(ml/** **m** ^**2**^ **)**					
Baseline	61.77 ± 21.81	59 (43.49 - 75.58)	46.29 ± 13.47	46.59 (34.44 - 55.74)	0.007
6 months	51.28 ± 16.63	47.64 (38.69 - 59.1)	42.07 ± 12.87	38.89 (33.58 - 48.67)	0.030
p-value**	0.001		0.173		
**LVESVi** **(ml/** **m** ^**2**^ **)**					
Baseline	27.32 ± 14.68	21.7 (16.56 - 34.86)	20.91 ± 9.81	18.54 (13.98 - 28.44)	0.115
6 months	21.17 ± 11.46	19.54 (13.55 - 24.99)	16.29 ± 7.99	13.56 (10.68 - 23.01)	0.074
p-value**	0.002		0.004		
**EF (%)**					
Baseline	57.63 ± 9.38	60.1 (54.8 - 63.5)	55.73 ± 10.15	55.45 (49.45 - 59.83)	0.079
6 months	61.28 ± 8.98	61.7 (58.55 - 66.95)	61.27 ± 11.35	61 (53.5 - 70.2)	0.841
p-value**	0.037		0.005		
**LVMI (** **g/** **m** ^**2**^ **)**					
Baseline	135.3 ± 37.5	120.1 (108.4 - 160.2)	137.46 ± 47.76	123.13 (97.5 - 173.89)	0.877
6 months	119.5 ± 36.8	110.7 (96.5 - 128.0)	124.44 ± 44.55	112.53 (98.75 - 155)	0.588
p-value**	0.002		0.537		
**LVMI/** **LVDVI**					
Baseline	2.49 ± 0.81	2.35 (1.84 - 2.96)	3.48 ± 1.7	3 (2.05 - 4.4)	0.038
6 months	2.46 ± 0.68	2.56 (1.9 - 2.92)	3.01 ± 1.53	2.82 (1.81 - 3.65)	0.334
p-value**	0.557		0.424		
**LAVI** **(ml/** **m** ^**2**^ **)**					
Baseline	39.94 ± 14.27	37.27 (31.15 - 47.99)	48.42 ± 14.81	47.5 (35.36 - 57.14)	0.008
6 months	38.16 ± 11.9	36.17 (30.19 - 45.24)	40.99 ± 12.7	42.63 (29.44 - 50)	0.425
p-value**	0.465		0.006		

LVDVI = left ventricular end-diastolic volume index; LVSVI = left ventricular end-systolic volume index; EF = ejection fraction; LVMI = left ventricular mass index; LAVI = left atrial volume index; *Mann–Whitney test; **Wilcoxon test.

At 6 months follow-up, 5 TAVI patients had died, 3 during hospitalization for TAVI, and 2 after hospital discharge from non-cardiovascular causes. There were no deaths in the SAVR group.

### Changes in LV remodeling and functional class after aortic valve replacement

**Figure 1:**
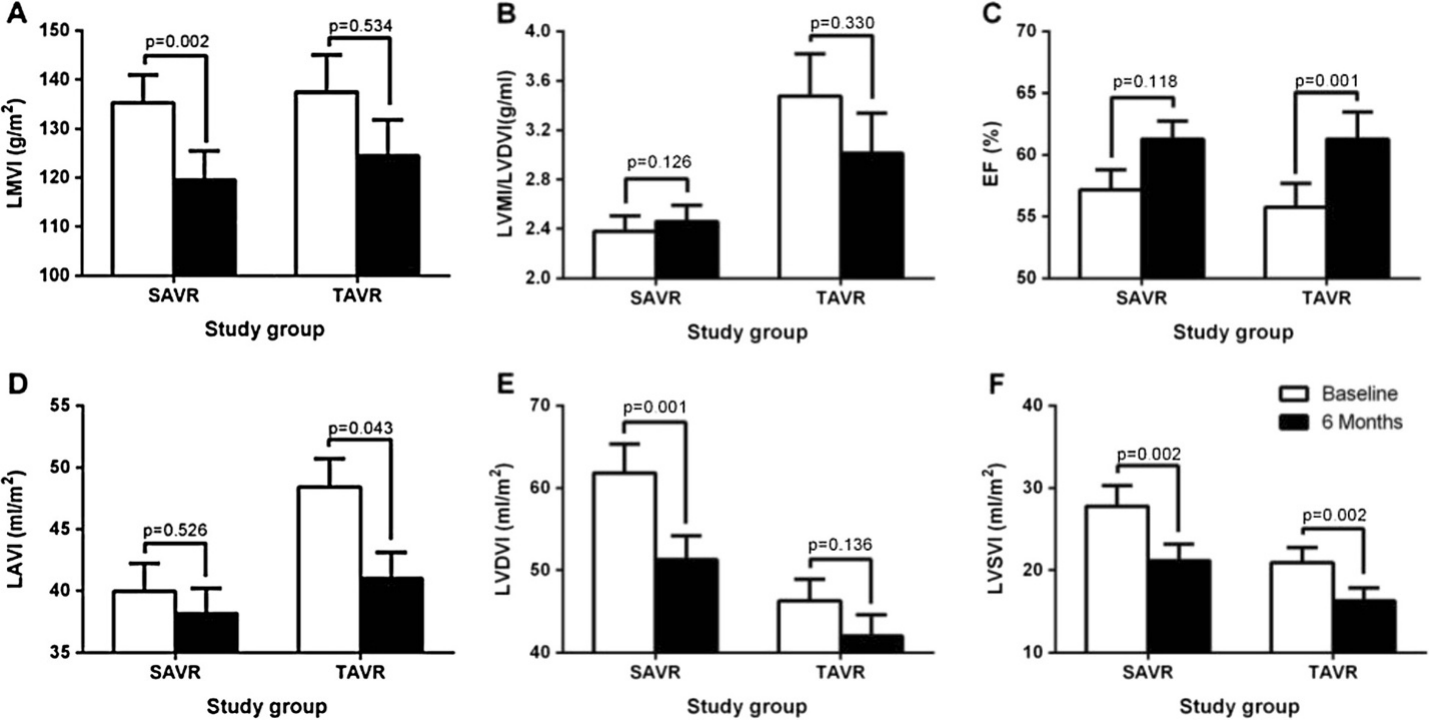
**Six months changes in parameters of remodeling in TAVI and SAVR groups.** **A**- change in left ventricular mass index (LVMI); **B**- change in ratio LVMI/left ventricular end-diastolic volume index (LVDVI); **C**- change in ejection fraction (EF); **D**- change in left atrial volume index (LAVI); **E**- change in LVDVI; **F**- change in left ventricular end-systolic volume index (LVSVI).

At 6 months (Table 2), TAVI patients had a higher effective orifice area index (EAOI) and lower transprosthetic maximal velocity and mean gradient, as well as a greater absolute increase in EAOI. Patient-prosthesis mismatch (PPM) was more frequent in the SAVR patients and there were no severe PPM cases in the TAVI group. There was a significant increase in ejection fraction (EF) in both groups and, when considering LV remodeling (Table 3), although there was a decrease in LV mass index (LVMI) and LV diastolic volume index (LVDVI) in both groups, only in SAVR patients this decrease was significant when compared with baseline values (Figure 1).

The presence of patient-prosthesis mismatch had no correlation with changes in LVMI, LVDVI, LV end-systolic volume index (LVSVI), or LA volume index (Additional file 1: Table S1). Moreover, EAOI increase was not related to final LVMI (r = 0.082, p = 0.512), LVDVI (r = 0.015, p = 0.925), LVSVI (r = 0.154, p = 0.331), or LAVI (r = 0.187, p = 0.143).

**Figure 2:**
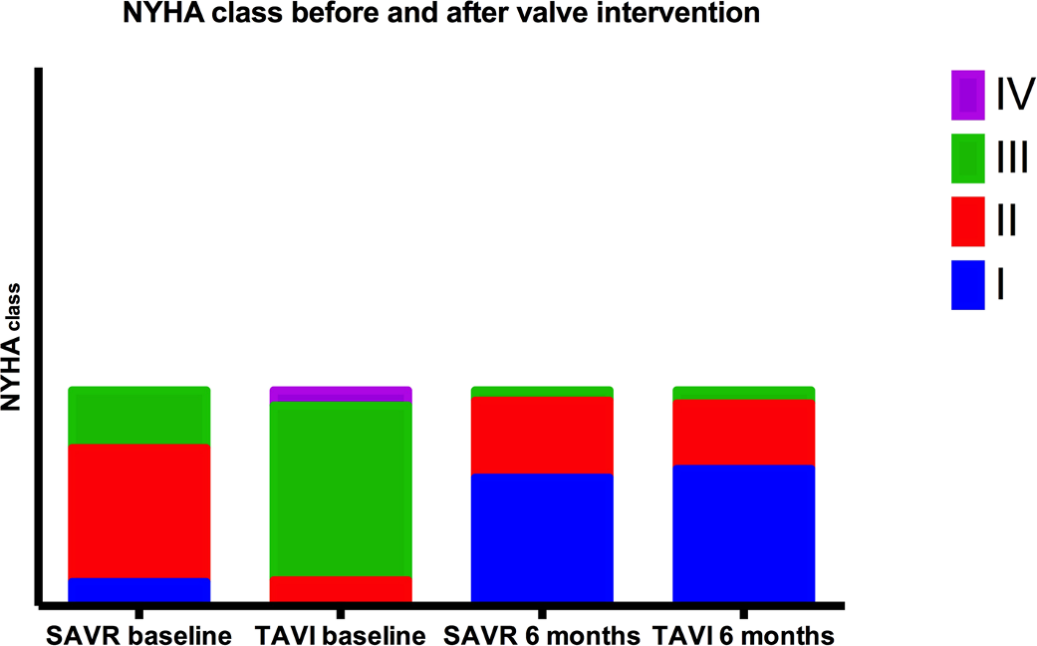
**Six months changes in NYHA class in TAVI and SAVR groups.**

After intervention, NYHA class was better in 30 (71.4%) TAVI patients compared with 22 (48.8%) SAVR patients (p = 0.001, Figure 2). Patients exhibiting a better NYHA class were more likely to have no PPM (81.6% vs 60.0%, p = 0.049) and a greater relative increase in EAOI (163.2% (P25-75: 118.9-234.8) vs 103.0% (P25-75: 52.1-170.2); p = 0.030). In univariate analysis, older age, baseline NYHA class ≥ III, a higher increase in EAOI and TAVI procedure were related with functional class improvement (Table 4). Moreover, we found no correlation between functional class improvement and parameters of LV reverse remodeling like the decrease in LV volumes or mass, or improvement in diastolic function suggested by the decrease in left atrial volume and E/e’ (Table 4). After a stepwise logistic multivariate regression analysis, the only independent predictors of NYHA class improvement were initial NYHA class ≥ III (OR 8.81, CI: 2.13-36.52; p = 0.003) and relative increase in EAOI ≥ 105% (OR 3.87, CI: 1.02-14.70; p = 0.04).

**Table 4:** **Univariate and multivariate analysis of clinical and echocardiographic determinants of NYHA class improvement**

NYHA improvement	Univariate analysis		Multivariate analysis	
	OR(95% CI)	p		p
**Female gender** **[n(%)]**	0.92 (0.31-2.67)	0.87		
**Age** **(years)**	1.09 (1.01-1.17)	0.02		
**ΔEF (%)**	0.99 (0.94-1.04)	0.62		
**ΔLVMI** **(g/** **m** ^**2**^ **)**	0.99 (0.97-1.01)	0.49		
**ΔLAVI** **(ml/** **m** ^**2**^ **)**	1.01 (0.99-1.02)	0.38		
**ΔLVDVI** **(ml** **/m** ^**2**^ **)**	0.98 (0.94-1.02)	0.28		
**ΔLVSVI** **(ml/** **m** ^**2**^ **)**	0.99 (0.93-1.05)	0.64		
**ΔEAOI (%)**	8.81 (0.90-86.10)	0.06	3.87 (1.02-14.70)	0.004
**ΔE/** **e’**	1.01 (0.94-1.08)	0.85		
**Hypertension [** **n(%)]**	1.70 (0.60-5.15)	0.35		
**Diabetes Mellitus** **[n(%)]**	0.48 (0.15-1.48)	0.20		
**COPD** **[n(%)]**	2.55 (0.75-8.66)	0.14		
**PAD** **[n(%)]**	0.80 (0.22-2.96)	0.74		
**NYHA class** ≥ **III** **[n(%)]**	11.33 (2.93-43.78)	<0.001	8.81 (2.13-36.52)	0.003
**TAVI [** **n(%)]**	7.08 (1.86-27.04)	0.004		
**Aortic regurgitation 6m [** **n(%)]**	2.35 (0.69-8.03)	0.17		

LVDVI = left ventricular end-diastolic volume index; LVSVI = left ventricular end-systolic volume index;; LVMI = left ventricular mass index; LAVI = left atrial volume index; Δ EF = baseline- 6 months ejection fraction; Δ LVDVI = baseline- 6 months LVDVI; Δ LVSVI = baseline- 6 months LVSVI; Δ LVMI = baseline- 6 months LVMI; Δ LAVI = baseline- 6 months LAVI; Δ EAOI = relative increase in EAOI at 6 months; Δ E/e’ = baseline- 6 months E/e’; COPD = chronic obstructive pulmonary disease; PAD = peripheral artery disease; TAVI = transcatheter aortic valve implantation.

Paravalvular regurgitation was more frequent after TAVI (59% vs 6.7%, p < 0.001), mostly of mild degree. Only 5 TAVI patients (5.8%) had moderate aortic regurgitation at six months. Paravalvular regurgitation had no correlation with the variation of indexed LVM, LVDV or LVSV (Additional file 2: Table S2). NYHA class improvement was similar in patients with and without paravalvular regurgitation (83.3% vs 66.7%, p = 0.241).

## Discussion

In this study we found that, at 6 months, TAVI patients had a better hemodynamic result and greater clinical improvement than those submitted to SAVR, but LV reverse remodeling was of a less significant degree than in SAVR patients.

### Reverse remodeling after aortic valve replacement

At 6 months follow-up, TAVI patients had a more favorable hemodynamic performance but the decrease in LVMI and LV volumes, although showing the same trend as SAVR patients, was less extensive.

A greater decrease in transvalvular gradients and increase in effective orifice area was seen after TAVI. It would be expected that patients undergoing this procedure had faster remodeling if load was the most important determinant of mass regression, but in our study EAOI increase was not associated with changes in LV mass and volumes after AVR. We can speculate that the baseline differences we have found, with older age and more comorbidities in TAVI patients, could have contributed to this result. Moreover, TAVI patients were “sicker”, with worse diastolic dysfunction and worse functional class, despite similar severity of AS and EF, possibly due to longer time of LV overload exposure. This could explain a restricted ability of the myocardium to recover from pre-intervention changes.

Various groups have focused on the hemodynamic and structural effects of TAVI [10, 15–18] with consistent results in afterload reduction and symptomatic improvement, but with conflicting results on reverse remodeling. Previous reports comparing the impact of TAVI and SAVR on LV remodeling addressed heterogeneous populations, including patients with coronary artery disease, different levels of EF, and several types of aortic prosthesis, including mechanical, stented and stentless bioprosthesis [9, 11, 19].

Clavel et al. [20] compared hemodynamic performances of TAVI or SAVR with stentless and stented bioprosthesis. At 6 to 12 months the increase in EAOI and reduction in transvalvular gradients in TAVI patients was similar to that obtained with stentless bioprosthesis and there was a clear advantage of TAVI in preventing PPM in patients with small annulus (≤20 mm). However, data on clinical improvement or reverse remodeling is absent.

Fairbairn et al. [19], using cardiac magnetic resonance imaging, showed a decrease in LVMI and indexed LV systolic volume at 6 months in both groups, but only SAVR patients had a decrease in indexed LV diastolic volume. The authors considered that the smaller reduction in LV end-diastolic volume post-TAVI could be related to a greater burden of coronary artery disease in these patients. In our study we found that, at 6 months, only SAVR patients had a significant decrease in LV mass and LV end-diastolic volume. Since we excluded patients with coronary artery disease, the absence of significant remodeling post-TAVI at this time point could indicate the existence of irreversible disease. Constantino et al. [18] compared reverse remodeling 2 months after TAVI and SAVR, and concluded that there was a more significant reduction in LVMI and relative wall thickness (RWT) in TAVI patients. These results are conflicting with ours, but were obtained in an earlier time point and the lack of adjustment for differences in baseline LVMI could have influenced results. Moreover, the authors found that these structural changes were paralleled by reduction in estimated filling pressures after TAVI. We also found a reduction in E/e´ after TAVI, but this occurred in absence of favorable remodeling and had no correlation with clinical improvement. Its association with prognosis is yet to be seen.

In the Cohort A of the PARTNER trial, LV diastolic volume was higher in TAVI patients in the first year of follow-up, but these differences were no longer present at two years. LV mass regression was faster in the SAVR group, although there were no significant differences after 6 months [11]. These results are similar to those reported in our study, suggesting that reverse remodeling can also occur in TAVI patients, but the process is slower than after SAVR, even after matching for age and major comorbidities.

Aortic regurgitation after TAVI, mostly paravalvular, is a common event [2, 21] and has come to our attention because of its impact on mortality. Post-procedural moderate to severe AR increased in-hospital mortality in comparison with no or only mild AR [21] and, in the randomized PARTNER trial, there was a positive correlation between AR severity and long-term mortality [22]. The pathophysiology underlying this increase is mortality is unclear. It has been speculated that significant paravalvular aortic regurgitation can overload the LV and impair reverse remodeling [23], therefore worsening prognosis. In our study, the presence of paravalvular aortic regurgitation had no correlation with the variation in LV volumes or mass. Once only 5 patients had moderate regurgitation, the lack of association with ventricular remodeling could be due to the small sample size. Longer follow-up and larger numbers are needed to take any definite conclusion on its impact in remodeling.

### Predictors of clinical improvement

In our sample, the increase in EAOI to more than the double was a strong predictor for clinical improvement, independent of changes in parameters of reverse remodeling. Conversely, the presence of patient-prosthesis mismatch (PPM) was correlated with impaired improvement in NYHA class.

Several studies reported that PPM is an independent predictor of cardiac events after AVR [24, 25] while others failed to demonstrate a significant impact on outcomes [26, 27].

Some authors found that persistent PPM results in less regression [28, 29] but even patients with PPM or small prosthesis can have significant reduction in LVM [30, 31]. The extent of regression is largely dependent on the extent of EOA increase after AVR [32]. Given the curvilinear relation of indexed EOA and transprosthetic gradients the degree of regression seems to be dependent on the original and final positions of an individual patient on the indexed EOA-gradient curve [33]. Although we found no correlation between PPM and impaired LVMI regression, we did find that the increase in valve area to more than twice the initial value was crucial for clinical improvement. This can be particularly important in elderly patients whose main concern is the achievement of a better quality of life.

Finally, as expected, patients who were in worse NYHA class before intervention more frequently experienced a clinical improvement. Using NYHA class to evaluate clinical improvement, although extensively used, has limitations and it is easier to demonstrate an improvement when a patient is class III/IV than NYHA class II/I. This fact can also help to explain the better improvement in the clinical status of patients undergoing TAVI, as they were in worse NYHA class than SAVR patients, before the intervention.

### Limitations

Since this was an observational study, we were not able to match patients for age, comorbidities or prosthesis size. These factors were considered in the multivariate model used for prediction of clinical improvement, but the authors recognize that, although logistic multivariate analysis is commonly used, it can’t correct for all possible confounders.

In addition, we selected our population by excluding patients with concomitant coronary artery disease and significant associated valvular disease to reduce introduction of further bias. The limited number of patients included in this analysis has limited statistical power to detect small differences between groups.

The evaluation of functional improvement using NYHA class is subjective and a more objective method, like six-minute walk test, could have allowed a quantitative assessment. Nevertheless, in clinical practice, NYHA class is the most widely use classification of function status and has been proven useful over the years.

## Conclusions

At six months after aortic valve intervention, better hemodynamic result was seen after TAVI, but LV reverse remodeling was of a less significant degree than after SAVR. Older age, comorbidities and the existence of a more extensive myocardial disease, as suggested by worse diastolic dysfunction and worse functional class in TAVI patients, despite similar severity of AS and EF, could explain a restricted ability of the myocardium to recover even after load relief. Moreover, six months may be too early to draw definitive conclusions, namely regarding the consequences of paravalvular aortic regurgitation.

Mid-term clinical improvement was strongly related to the increase in EAOI and had no association with LV remodeling parameters. Thus, doubling the initial aortic valve area seems to be a key point to achieve clinical improvement after valve replacement, a particularly important endpoint in the elderly.

This study raises some important new questions but longer follow-up and large-scale randomized trials are needed to confirm these results.

## Acknowledgements

This work was supported by the Cardiovascular R&D Center, through grants from the Portuguese Foundation for Science and Technology (PEst-C/SAU/UI0051/2011, EXCL/BIM-MEC/0055/2012 and HMSP-ICS/0007/2012; partially funded by FEDER through COMPETE) and from the European Commission (FP7-Health-2010; MEDIA-261409).

## Electronic supplementary material

Additional file 1: Table S1: Correlation of the presence of patient prosthesis mismatch (EAOI ≤ 0.85 cm^2^) and changes in indexed 3D volumes and left ventricular mass. (DOC 35 KB)

Additional file 2: Table S2: Correlation of the presence of aortic regurgitation at 6 months and changes in indexed 3D volumes and left ventricular mass. (DOC 36 KB)

## Abbreviations

SAVR: Surgical aortic valve replacement
TAVI: transcatheter aortic valve implantation
BSA: body surface area
COPD: chronic obstructive pulmonary disease
PAD: peripheral artery disease
DM: diabetes mellitus
HT: arterial hypertension
NYHA: New York Heart Association
AV: aortic valve
AVA: aortic valve area
EAOI: effective orifice area index
PPM: patient-prosthesis mismatch
IVRT: Isovolumetric relaxation time
LVDVI: left ventricular end-diastolic volume index
LVSVI: left ventricular end-systolic volume index
LVMI: left ventricular mass index
LAVI: left atrial volume index.
