# Supplementary material for: Determinants of clinical improvement after surgical replacement or transcatheter aortic valve implantation for isolated aortic stenosis
Source: Cardiovasc Ultrasound. 2014 Oct 6;12:41. doi: 10.1186/1476-7120-12-41 (PMC4197280; doi:10.1186/1476-7120-12-41)
Supplement: Supplementary file 1 — Additional file 1: Table S1: Correlation of the presence of patient prosthesis mismatch (EAOI ≤ 0.85 cm2) and changes in indexed 3D volumes and left ventricular mass. (DOC 35 KB) [file 12947_2014_536_MOESM1_ESM.doc]

**Additional file 1: Table S1**. Correlation of the presence of patient prosthesis mismatch (EAOI≤0.85 cm^2^) and changes in indexed 3D volumes and left ventricular mass.

| **Patient prosthesis mismatch (PPM)** | **EAOI>0.85 cm^2^** | **EAOI≤0.85 cm^2^** |  |
| --- | --- | --- | --- |
|  | **Me (P25-P75)** | **Me (P25-P75)** | **p** |
| **3D ΔLVMI (g/m^2^)** | 9.8 (P25-75:-24.5-27.1) | 11.0 (P25-75:-4.2-24.8) | p=0.619 |
| **3D ΔLAVI (ml/m^2^)** | 14.9 (P25-75:-5.8-34.1) | 7.2 (P25-75:-20.2-30.2) | p=0.519 |
| **3D ΔLVDVI (ml/m^2^)** | 12.6 (P25-75:0.4-24.8) | 11.0 (P25-75:-5.0-20.1) | p=0.315 |
| **3D ΔLVSVI (ml/m^2^)** | 8.5 (P25-75:2.6-15.7)) | 4.0 (P25-75:0.0-12.4) | p=0.167 |

LVDVI= left ventricular end-diastolic volume index; LVSVI= left ventricular end-systolic volume index; LVMI=left ventricular mass index; LAVI= left atrial volume index; Δ LVDVI = baseline- 6 months LVDVI; Δ LVSVI= baseline- 6 months LVSVI; Δ LVMI= baseline- 6 months LVMI; Δ LAVI= baseline- 6 months LAVI
