# Supplementary material for: Determinants of clinical improvement after surgical replacement or transcatheter aortic valve implantation for isolated aortic stenosis
Source: Cardiovasc Ultrasound. 2014 Oct 6;12:41. doi: 10.1186/1476-7120-12-41 (PMC4197280; doi:10.1186/1476-7120-12-41)
Supplement: Supplementary file 2 — Additional file 2: Table S2: Correlation of the presence of aortic regurgitation at 6 months and changes in indexed 3D volumes and left ventricular mass. (DOC 36 KB) [file 12947_2014_536_MOESM2_ESM.doc]

**Additional file 2: Table S2**. Correlation of the presence of aortic regurgitation at 6 months and changes in indexed 3D volumes and left ventricular mass.

|  | **Without AR 6m** |  | **With AR 6m** |  |
| --- | --- | --- | --- | --- |
|  | **Me (P25-P75)** |  | **Me (P25-P75)** | **p** |
| **3D ΔLVMI (g/m^2^)** | 10.99 (-7.2 - 24.53) |  | 6.4 (-24.52 - 37.22) | 0.793 |
| **3D ΔLAVI (ml/m^2^)** | 15.52 (-19.84 - 33.51) |  | 10.54 (-7.42 - 33.2) | 0.928 |
| **3D ΔLVDVI (ml/m^2^)** | 11.85 (1.15 - 24.02) |  | 1.47 (-11.21 - 20.6) | 0.177 |
| **3D ΔLVSVI (ml/m^2^)** | 5.4 (2.09 - 12.74) |  | 7.88 (1.77 - 14.96) | 0.940 |

LVDVI= left ventricular end-diastolic volume index; LVSVI= left ventricular end-systolic volume index; LVMI=left ventricular mass index; LAVI= left atrial volume index; Δ LVDVI = baseline- 6 months LVDVI; Δ LVSVI= baseline- 6 months LVSVI; Δ LVMI= baseline- 6 months LVMI; Δ LAVI= baseline- 6 months LAVI
